# Machine learning‐based classification of aMCI and non‐aMCI using gait and MRI‐based characteristics

**DOI:** 10.1002/alz.086713

**Published:** 2025-01-03

**Authors:** Yeo Jin Kim, Ingyu Park, Sang‐Kyu Lee, Hui‐Chul Choi, Moo‐Eob Ahn, Ohk‐Hyun Ryu, Daehun Jang, Unjoo Lee

**Affiliations:** ^1^ Kangdong Sacred Heart Hospital, Seoul, Seoul Korea, Republic of (South); ^2^ Hallym University, Chuncheon, Gangwon‐do Korea, Republic of (South); ^3^ Hallym University College of Medicine, Chuncheon, Gangwon‐do Korea, Republic of (South)

## Abstract

**Background:**

In patients with mild cognitive impairment (MCI), the presence or absence of memory deficits is associated with divergent clinical presentations, etiologies, and prognostic outcomes. These differences may also manifest in additional neurologic signs beyond cognitive impairments and are often reflected in distinct magnetic resonance imaging (MRI) profiles. Gait is one of the clinical characteristics that reflects brain function along with cognitive function. Therefore, we aim to distinguish between amnestic MCI (aMCI) and non‐aMCI (naMCI) using gait and MRI‐based biomarkers.

**Method:**

Eighty patients diagnosed with MCI from three dementia centers in Gangwon‐do, Korea were recruited for this study. We defined MCI as having a Clinical Dementia Rating global score of 0.5, with a memory domain score at or above 0.5. All participants underwent the Consortium to Establish a Registry for Alzheimer’s Disease (CERAD) assessment for cognitive evaluation, gait assessment using GAITRite electronic walkway system and brain MRI. Based on CERAD, individuals with a verbal memory delayed recall score at or below ‐1.0 standard deviation from the norm were classified as aMCI, while those with scores higher than this threshold were categorized as naMCI. We trained a machine learning model using gait and MRI data parameters.

**Result:**

The CNN resulted in the best classifier performance in separating aMCI from naMCI; its performance was maximized when only feature patterns that included GAIT set were used. (accuracy = 0.99 ± 0.04) The standard deviation of stride velocity and the standard deviation of stride length were the strongest predictors. Subsequently, machine learning analyses using the GAIT + WM data set (accuracy = 0.96±0.07) and the GAIT + GM dataset (0.95±0.08) demonstrated high accuracy.

**Conclusion:**

Machine learning using GAIT data set achieved the highest accuracy in distinguishing between aMCI and naMCI.

